# Association Between Circulating Retinol-Binding Protein 4 and Adverse Cardiovascular Events in Stable Coronary Artery Disease

**DOI:** 10.3389/fcvm.2022.829347

**Published:** 2022-03-17

**Authors:** Ke Qian, Xin Yan, Cheng Xu, Yijia Fang, Moshuang Ma

**Affiliations:** ^1^Department of Emergency, Liyang People's Hospital, Liyang, China; ^2^Department of General Surgery, Liyang People's Hospital, Liyang, China

**Keywords:** retinol-binding protein 4, coronary artery disease, major adverse cardiovascular events, Chinese, cohort

## Abstract

**Background:**

The predictive role of retinol-binding protein 4 (RBP4) in the adverse prognosis of patients with stable coronary artery disease (CAD) has not been well-defined. We thus conducted this cohort study to investigate the association between circulating RBP4 level and major adverse cardiovascular events (MACEs) in Chinese patients with stable CAD.

**Methods:**

Patients with stable CAD and serum RBP4 concentration measurement at admission between July 2012 and January 2015 were included. The primary outcome in this study was incident MACEs, which included acute coronary syndrome, heart failure, stroke, peripheral vascular disease, and cardiovascular death. Cox proportional hazards regression was adopted to investigate the association between RBP4 and the incidence of MACEs.

**Results:**

A total of 840 patients with stable CAD were analyzed. The mean age of patients was 61.2 ± 15.9 years, and 56.1% of them were men. After a median follow-up of 2.3 years, 129 MACEs were observed. Compared to participants exposed to the first quartile of serum RBP4 level, those in the second, the third, and the fourth quartiles had associated hazard ratios (HRs) of 2.38 [95% confidence interval (CI): 1.33–4.26], 2.35 (95% CI: 1.31–4.21), and 2.27 (95% CI: 1.28–4.04) after adjusted for confounders, respectively. Every 5 μg/ml increment in serum RBP4 concentration was associated with an adjusted HR of 1.13 (95% CI: 1.05–1.22) for the occurrence of MACEs. Subgroup analyses suggested no significant modifying effects of baseline characteristics for the association between RBP4 and MACEs in patients with stable CAD.

**Conclusion:**

Our finding suggested that the higher circulating RBP4 level was significantly associated with an increased risk of MACEs in patients with stable CAD.

## Introduction

Cardiovascular disease (CVD) is the leading cause of morbidity and mortality worldwide, and nearly 330 million Chinese population were affected in 2019 (1). As reported earlier, although cardiovascular mortality has declined in some developed countries, this data still tend to rise in low- and middle-income countries, including China (2). The major CVD risk factors have been well-identified in epidemiological studies. Among the potentially modifiable risk factors, obesity and adipose tissue are regarded as a promising target in the prevention of CVD (3). Coronary artery disease (CAD) is one of the most common entities of CVD profiles. Considering the relevance of diagnosis and treatment, increasing studies have focused on identifying blood biomarkers for CAD (4).

Retinol-binding protein 4 (RBP4) is an adipokine, which is mainly synthesized and secreted in the liver and adipose tissue (5). In adipose and vascular tissues, RBP4 is involved in the progression of insulin resistance by regulating the immunity and inflammatory reaction (6, 7). Previous experimental research has found that RBP4 could impair insulin-stimulated glucose uptake and promote inflammatory damage in cardiac myocytes (8). In recent years, a number of studies have observed significant correlations between RBP4 and the risk of CAD development (9–13). In the Nurses' Health Study, the higher circulating and total RBP4 levels were found to be significantly associated with an increased risk of CAD (11). Plasma RBP4 level also increases in line with the coronary lesion complexity (9) and the number of narrowed coronary arteries (10). Besides, positive associations were also observed between RBP4 and traditional CVD risk factors, including dyslipidemia (14), hypertension (15), metabolic syndrome (16), and coronary artery calcification (17). However, there are few studies investigating the predictive role of RBP4 in the adverse prognosis in patients with stable CAD.

Therefore, we conducted this cohort study to examine the association between circulating RBP4 level and major adverse cardiovascular events (MACEs) in Chinese patients with stable CAD.

## Methods

### Selection of Patients

All consecutive patients diagnosed with stable CAD (*n* = 1,356) in our center from July 2012 to January 2015 were retrospectively screened. The exclusion criteria were as follows: (1) without available data for RBP4 concentration (*n* = 272); (2) prior history of cancer (*n* = 8); (3) infectious disease (*n* = 55); (4) heart failure (HF) (*n* = 93); and (5) refusing participation (*n* = 71). Furthermore, 17 patients were further excluded due to loss to follow-up. As a result, a total of 840 patients were included in the final analysis. The flowchart of patients' selection was shown in Supplementary Figure 1. The diagnosis of stable CAD was defined according to the American College of Cardiology guidelines (18). All patients were asked to sign an informed consent form before participating in this study. This study was approved by the Ethics Committee of Liyang People's Hospital. The identified information was removed before the data was released.

### Serum RBP4 Measurement

Fasting blood samples were prospectively obtained with anticoagulants from all patients at their first admission. Blood specimens were immediately cooled on ice and subsequently centrifuged at 4,500 rpm for 8 min at 4°C. The supernatant after centrifugation was extracted and stored at 80°C until further use. The serum RBP4 concentrations were subsequently measured using an ELISA method (AdipoGen, Seoul, Korea) in all available samples, according to the manufacturer's protocol, and compared with purified human RBP4 standards. The minimum detection limit was 1 ng/ml. All samples were analyzed in duplicate. The average intra-assay and interassay coefficients of variation were 2.32–8.77 and 2.95–9.27%, respectively.

### Covariates' Collection

Information on participants' demographic characteristics, comorbidities, and medical history were collected by a standard self-administered questionnaire and further verified by objective imaging modalities, blood tests, and medication lists from electrical medical records. Height and weight were measured using an anthropometer (KN-5000a, Nakamura, Tokyo, Japan) with participants wearing light indoor clothing and no shoes. Body mass index (BMI) was calculated according to the following equation: BMI (kg/m^2^) = body weight (kg)/height squared (m^2^). We recorded comorbid conditions, such as hypertension, diabetes mellitus, and chronic kidney disease (CKD), given that they were potentially related to circulating RBP4 level (19). In addition, medication usage, including beta-blocker, angiotensin-converting enzyme inhibitor (ACEI)/angiotensin receptor blocker (ARB), and statins, was also assessed.

### Study Outcome and Follow-Up

The primary outcome of interest in this study was incident MACEs, which comprised acute coronary syndrome, HF, stroke, peripheral vascular disease, or cardiovascular death. All patients were followed up *via* clinic visits or by phone. The incident events were confirmed by objective information from medical records. The follow-up period began at enrollment and continued until the occurrence of MACES, last visit, or December 31, 2020, whichever came first.

### Statistical Methods

All statistical analyses were conducted using R 3.3.2 (R Core Team, Vienna, Austria), and two-sided *p*-value of <0.05 was considered statistically significant. Continuous data were expressed as mean ± SD, and group comparisons were performed using the one-way ANOVA. Categorical variables were depicted as number and percentage, and group differences were compared by the Pearson's chi-square test. Cox proportional hazards regression was adopted to prospectively investigate the association between RBP4 and the incidence of MACEs, as well as its individual components. The independent variable of RBP4 was treated both as continuous variable and categorical variable (i.e., categorizing RBP4 into quartiles and selecting the first quartile as the reference group) to explore the relationship between RBP4 and the risk of MACEs in patients with stable CAD. Three models were developed, namely, Model 1 was adjusted for age (years), sex (male or female), BMI (kg/m^2^, continuous), and smoking (yes or no); Model 2 further adjusted for hypertension (yes or no), diabetes mellitus (yes or no), and CKD (yes or no); and Model 3 comprised Model 2 plus further adjustment for medicine use, including beta-blocker (yes or no), statins (yes or no), or ACEI/ARB (yes or no). Subgroup analyses were conducted with stratification by age, sex, BMI, smoking, hypertension, diabetes mellitus, and CKD to examine the robustness of the association between circulating RBP4 level and MACEs in patients with stable CAD.

## Results

### Characteristics of Patients

Among the 840 patients with stable CAD, the mean age was 61.2 ± 15.9 years, and 56.1% of them were men. The mean level of serum RBP4 in all patients was 35.8 ± 11.7 μg/ml. Notably, 25.5% of patients had a history of ST-segment elevation myocardial infarction (STEMI), while 44.3% of patients had a history of non-ST-segment elevation myocardial infarction (NSTEMI), and 8.6% of patients had unstable angina. Regarding prior invasive treatment, 70.2 and 5.0% of patients ever received the percutaneous coronary intervention and coronary artery bypass grafting (CABG), respectively. The comparisons of general characteristics between patients in different quartiles of circulating RBP4 concentrations are presented in Table 1. Among them, except for prior CABG, no significant difference was observed in demographic characteristics, comorbidities, and medication usage. After a median follow-up of 2.3 years, 129 patients with stable CAD suffered from MACEs, comprising 45 acute coronary syndromes, 33 HFs, 18 strokes, seven peripheral vascular diseases, and 26 cardiovascular deaths. Compared to patients without MACEs, those with MACEs were older (61.3 ± 16.0 vs. 64.9 ± 15.4 years, *p* = 0.020) and had a higher level of BMI (26.1 ± 4.6 vs. 27.8 ± 4.6 kg/m^2^, *p* < 0.001) and RBP4 (35.2 ± 11.8 vs. 39.2 ± 10.3 μg/ml, *p* < 0.001). Moreover, they were more likely to have a history of STEMI and CKD but take less statins or ACEI/ARB (Table 2).

**Table 1:** Baseline characteristics of patients according to circulating RBP4 concentration.

**Variables**	**Quartile 1 (*n* = 210)**	**Quartile 2 (*n* = 210)**	**Quartile 3 (*n* = 210)**	**Quartile 4 (*n* = 210)**	***P*-value**
	**(14.4–26.0 μg/ml)**	**(26.0–36.7 μg/ml)**	**(36.7–46.0 μg/ml)**	**(46.0–55.9 μg/ml)**	
Age, years, mean (SD)	62.6 ± 15.2	62.0 ±15.1	62.2 ± 16.3	60.7 ± 17.0	0.611
Female, *n* (%)	95 (45.2)	94 (44.8)	98 (46.7)	82 (39.0)	0.411
BMI, kg/m^2^, mean (SD)	25.8 ± 4.4	26.5 ± 4.6	26.3 ± 4.9	27.0 ± 4.7	0.080
Current smoker, *n* (%)	49 (23.2)	35 (16.7)	35 (16.7)	52 (24.8)	0.066
Current drinker, *n* (%)	34 (16.3)	26 (12.4)	30 (14.4)	38 (18.1)	0.400
**Comorbidity**, ***n*** **(%)**					
Prior history of MI	146 (69.5)	149 (71.0)	149 (71.0)	142 (67.6)	0.863
STEMI	53 (25.2)	43 (20.5)	55 (26.2)	63 (30.0)	0.165
NSTEMI	93 (44.3)	106 (50.5)	94 (44.8)	79 (37.6)	0.070
Prior history of UA	19 (9.0)	18 (8.6)	15 (7.1)	20 (9.5)	0.837
Prior PCI	156 (74.3)	146 (69.5)	151 (71.9)	137 (65.2)	0.213
Prior CABG	11 (5)	14 (6.7)	3 (1.4)	14 (6.7)	0.044
Hypertension	78 (37.1)	90 (42.9)	84 (40.0)	92 (43.8)	0.501
Diabetes mellitus	66 (31.4)	50 (23.8)	57 (27.1)	50 (23.8)	0.239
CKD	18 (8.6)	15 (7.1)	12 (5.7)	21 (10.0)	0.398
**Medications**, ***n*** **(%)**					
Beta-blocker	132 (62.9)	137 (65.2)	138 (65.7)	132 (62.9)	0.888
Statins	113 (53.8)	87 (41.4)	106 (50.5)	105 (50.0)	0.070
ACEI/ARB	134 (63.8)	141 (67.1)	129 (61.4)	135 (64.3)	0.681

*RBP4, retinol-binding protein 4; SD, standard deviation; BMI, body mass index; MI, myocardial infarction; STEMI, ST-segment elevation myocardial infarction; NSTEMI, non-ST-segment elevation myocardial infarction; UA, unstable angina; PCI, percutaneous coronary intervention; CABG, coronary artery bypass grafting; CKD, chronic kidney disease; ACEI, angiotensin-converting enzyme inhibitor; ARB, angiotensin receptor blocker*.

**Table 2:** Differences in baseline characteristics between patients with and without MACEs.

**Variables**	**Without MACEs (*n* = 711)**	**With MACEs (*n* = 129)**	***P*-value**
Age, years, mean (SD)	61.3 ± 16.0	64.9 ± 15.4	0.020
Female, *n* (%)	405 (57.0)	66 (51.2)	0.247
BMI, kg/m^2^, mean (SD)	26.1 ± 4.6	27.8 ± 4.6	<0.001
RBP4, μg/ml, mean (SD)	35.2 ± 11.8	39.2 ± 10.3	<0.001
Current smoker, *n* (%)	147 (20.7)	24 (18.6)	0.636
Current drinker, *n* (%)	109 (15.4)	19 (14.7)	1.000
**Comorbidity**, ***n*** **(%)**			
Prior history of MI	492 (69.2)	94 (72.9)	0.404
STEMI	167 (23.5)	47 (36.4)	0.002
NSTEMI	325 (45.7)	47 (36.4)	0.051
Prior history of UA	58 (8.2)	14 (10.9)	0.314
Prior PCI	498 (70.0)	92 (71.3)	0.771
Prior CABG	34 (4.8)	8 (6.2)	0.496
Hypertension	296 (41.6)	48 (37.2)	0.382
Diabetes mellitus	186 (26.2)	27 (28.7)	0.588
CKD	48 (6.8)	18 (14.0)	0.011
Prior history of MI	492 (69.2)	94 (72.9)	0.466
**Medications**, ***n*** **(%)**			
Beta-blocker	466 (65.5)	73 (56.6)	0.058
Statins	369 (51.9)	42 (32.6)	<0.001
ACEI/ARB	470 (66.1)	69 (53.5)	0.008

*MACEs, major adverse cardiovascular events; BMI, body mass index; RBP4, retinol-binding protein 4; MI, myocardial infarction; STEMI, ST-segment elevation myocardial infarction; NSTEMI, non-ST-segment elevation myocardial infarction; UA, unstable angina; PCI, percutaneous coronary intervention; CABG, coronary artery bypass grafting; CKD, chronic kidney disease; ACEI, angiotensin-converting enzyme inhibitor; ARB, angiotensin receptor blocker*.

### Relationship Between Circulating RBP4 Level and MACEs

**Figure 1:**
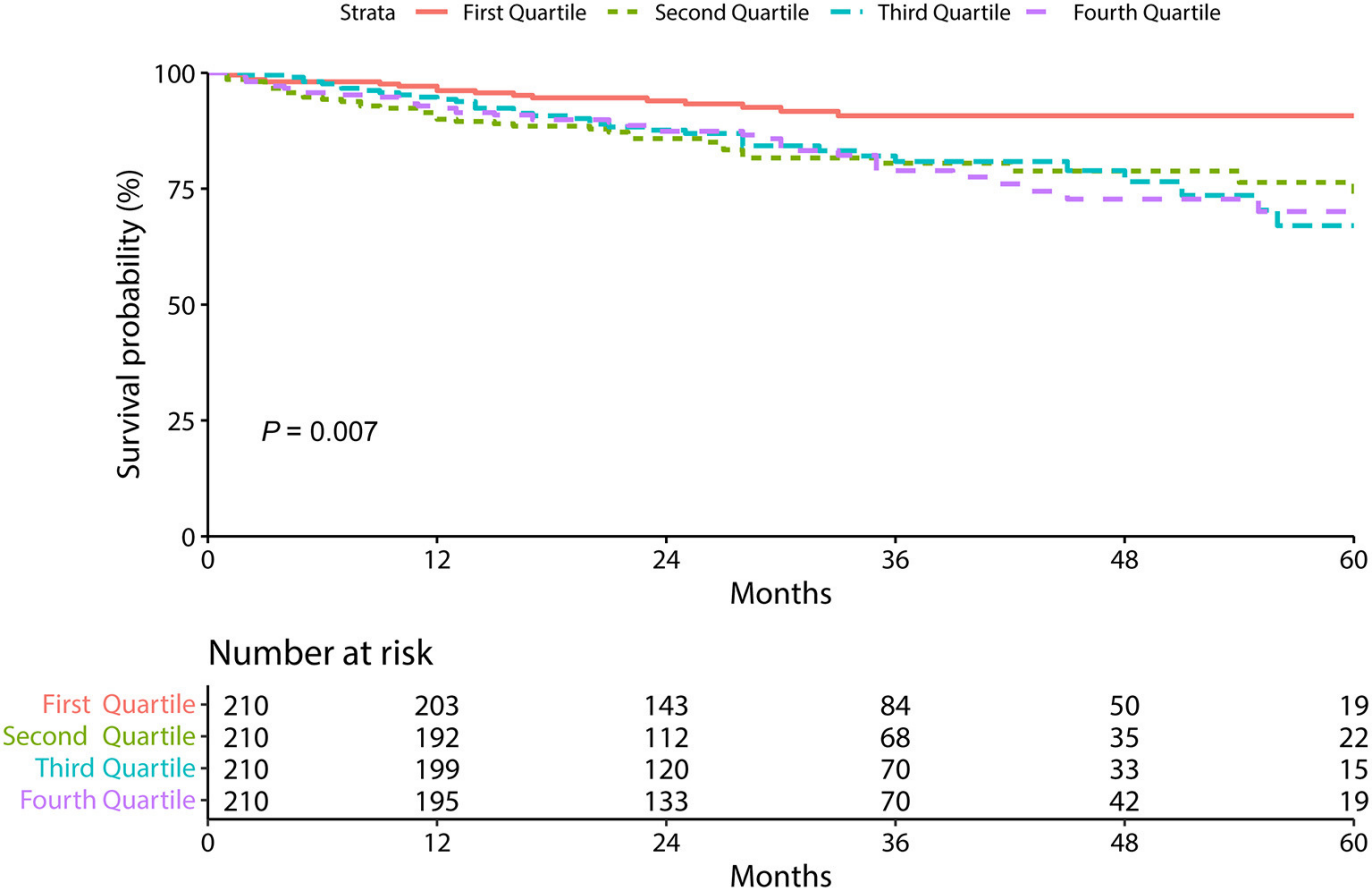
The Kaplan-Meier survival curves according to circulating retinol-binding protein 4 level.

The relationship between serum RBP4 level and MACEs in patients with stable CAD is presented in Table 3 and Figure 1. Three gradually adjusted models show consistent results. Compared to patients in the first quartile of serum RBP4 level (reference group), those exposed to the second, the third, and the fourth quartiles had associated hazard ratios (HRs) of 2.38 [95% confidence interval (CI): 1.33–4.26], 2.35 (95% CI: 1.31–4.21), and 2.27 (95% CI: 1.28–4.04), respectively, for MACEs development after adjustment for age, sex, BMI, smoking, hypertension, diabetes mellitus, CKD, and the medication usage. In addition, every 5 μg/ml increment in serum RBP4 level was associated with an adjusted HR of 1.13 (95% CI: 1.05–1.22) for MACEs in patients with stable CAD. Regarding the individual outcome of MACEs, serum RBP4 level was significantly associated with an increased risk of HR (1.18, 95% CI: 1.02–1.38) and showed a trend to increase the risk of acute coronary syndrome, stroke, peripheral vascular disease, and cardiovascular death, despite failing to reach statistical significance (Supplementary Figure 2).

**Table 3:** The associations of circulating RBP4 concentration and MACEs in patients with stable CAD.

	**Model 1**		**Model 2**		**Model 3**	
	**HR (95% CI)**	***P*-value**	**HR (95% CI)**	***P*-value**	**HR (95% CI)**	***P*-value**
Quartile 1 (14.4–26.0 μg/ml)	Ref		Ref		Ref	
Quartile 2 (26.0–36.7 μg/ml)	2.42 (1.36–4.30)	0.003	2.54 (1.42–4.53)	0.002	2.38 (1.33–4.26)	0.003
Quartile 3 (36.7–46.0 μg/ml)	2.35 (1.32–4.19)	0.004	2.49 (1.39–4.44)	0.002	2.35 (1.31–4.21)	0.004
Quartile 4 (46.0–55.9 μg/ml)	2.37 (1.34–4.19)	0.003	2.36 (1.33–4.19)	0.003	2.27 (1.28–4.04)	0.005
Trend test		0.008		0.008		0.013
Every 5 μg/ml increase	1.14 (1.05–1.22)	0.001	1.13 (1.05–1.22)	0.001	1.13 (1.05–1.22)	0.002

*Data are complete for all variables*.

*Model 1: adjusted for age, sex, body mass index, and smoking; Model 2: adjusted for age, sex, body mass index, smoking, hypertension, diabetes mellitus, and chronic kidney disease; Model 3: adjusted for age, sex, body mass index, smoking, hypertension, diabetes mellitus, chronic kidney disease, and the usage of beta-blocker, statins, or ACEI/ARB*.

*RBP4, retinol binding protein 4; MACEs, major adverse cardiovascular events; BMI, body mass index; CAD, coronary artery disease; HR, hazard ration; CI, confidence interval; Ref, reference; ACEI, angiotensin-converting enzyme inhibitor; ARB, angiotensin receptor blocker*.

### Subgroup Analyses

Table 4 depicts the results of subgroup analyses, which suggested that there are no significant modifying effects of age (*P*_*interaction*_ = 0.758), sex (*P*_*interaction*_ = 0.180), BMI (*P*_*interaction*_ = 0.749), smoking (*P*_*interaction*_ = 0.708), hypertension (*P*_*interaction*_ = 0.893), diabetes mellitus (*P*_*interaction*_ = 0.625), and CKD (*P*_*interaction*_ = 0.490) for the association between RBP4 and MACEs in patients with stable CAD.

**Table 4:** Subgroup analyses for the association between circulating RBP4 concentration and MACEs.

**Covariates**	**Events/total**	**HR (95% CI)**	***P*-value**	***P_***interaction***_***
**Sex**				0.758
Male	66/471	1.14 (1.02–1.27)	0.020	
Female	63/369	1.12 (1.00–1.25)	0.044	
**Age (years)**				0.180
<65	50/429	1.18 (1.03–1.34)	0.014	
≥65	79/411	1.08 (0.98–1.19)	0.141	
**BMI (kg/m** ^**2**^ **)**				0.749
<25	46/360	1.16 (1.02–1.32)	0.028	
≥25	83/480	1.13 (1.02–1.24)	0.014	
**Current smoking**				0.708
No	105/669	1.12 (1.03–1.22)	0.008	
Yes	24/171	1.17 (0.97–1.40)	0.094	
**Hypertension**				0.893
No	81/496	1.11 (1.01–1.22)	0.028	
Yes	48/344	1.75 (0.76–4.03)	0.188	
**Diabetes mellitus**				0.625
No	92/617	1.14 (1.04–1.25)	0.006	
Yes	37/223	1.15 (0.99–1.34)	0.067	
**CKD**				0.490
No	111/774	1.13 (1.04–1.23)	0.003	
Yes	18/66	1.00 (0.80–1.25)	0.991	

*The models were adjusted for age (except for the age-stratified analysis), sex (except for the sex-stratified analysis), BMI (except for the BMI-stratified analysis), smoking (except for the smoking-stratified analysis), hypertension (except for the hypertension-stratified analysis), diabetes mellitus (except for the diabetes mellitus-stratified analysis), CKD (except for the CKD-stratified analysis), and the usage of beta-blocker, statins, or ACEI/ARB. The HRs were calculated based on every 5 μg/ml increase of RBP4 level*.

*RBP4, retinol binding protein 4; MACEs, major adverse cardiovascular events; HR, hazard ration; CI, confidence interval; BMI, body mass index; CKD, chronic kidney disease; ACEI, angiotensin-converting enzyme inhibitor; ARB, angiotensin receptor blocker*.

## Discussion

In this cohort study, we first found that elevated circulating RBP4 level was significantly associated with an increased risk of MACEs in patients with stable CAD. This association remained after the adjustment of covariates and kept consistent among different subgroup analyses. We also assessed the specified association of RBP4 and individual components of MACEs and found that HF may primarily drive the positive association.

Epidemiological studies suggested that RBP4 might be involved in the pathogenesis of atherosclerosis. Elevated circulating RBP4 levels have been observed in subjects with previous clinical arteriosclerosis (20), subclinical arteriosclerosis (21), and CAD (9–12). Moreover, Kadoglou et al. found that RBP4 concentration, independent of symptoms existence, was significantly elevated in high-grade carotid stenosis compared to low-grade carotid stenosis (22). Similarly, Sun et al. also reported that elevated RBP4 was correlated with an increased risk of CAD and the severity of CAD quantified by the Gensini score in patients with subclinical hypothyroidism (23). These findings favor the rationality that RBP4 is a novel risk factor for the development of CAD and is associated with disease severity and subsequent adverse outcome. In our study, we first found that elevated circulating RBP4 level significantly increases the risk of MACEs in patients with stable CAD during follow-up. Regarding the individual outcome of MACEs, circulating RBP4 level was significantly associated with HF rather than cardiovascular death, which was different from a prior study that reported that total RBP4 level was a predictor of cardiac death in patients with stable CAD or acute coronary syndrome (9). Considering the similar circulating RBP4 level of patients with stable CAD in the two studies [35.8 ± 11.7 μg/ml vs. 34.74 (range 27.65–40.19) μg/ml], we speculated that different study populations are the main source of distinct findings in the view of the fact that patients with acute coronary syndrome are more likely to have a higher level of RBP4 than those with stable CAD (9).

The RBP4 was initially discovered as an adipocytokine, which is specifically bound to vitamin A (retinol) (24). RBP4 is known to be produced mainly by the liver in the past. The physiological function of RBP4 is to transport vitamin A to peripheral target tissues from the liver and acts as a major maintainer of circulating retinol levels (25). However, ~15% of circulating RBP4 was actually secreted from adipose tissue (26). Serum RBP4 levels are also found to be elevated in insulin-resistant mice and humans with obesity and diabetes (27). Then, a growing body of evidence has supported the involvement of RBP4 on obesity and insulin resistance in humans (28). The underlying mechanisms of RBP4 involved in the pathophysiology of CAD are still controversial. First, as mentioned earlier, elevated RBP4 level impairs insulin cascade signaling in muscle and visceral adipose tissue, contributing to the pathogenesis of insulin resistance, and both the incident obesity and type 2 diabetes (27) were the well-established risk factors for CAD and its severity (3). Second, RBP4 plays a pivotal role in the inflammation and mitochondrial dysfunction of endothelial cells, both of which significantly accelerate or modify atherogenesis in the coronary artery (7, 29). Third, elevated RBP4 in aortic atherosclerotic lesions from both human and mice could facilitate the formation of macrophage-derived foam cell in way of activating cholesterol uptake, thus accelerating the progression of atherosclerosis (30). Given the potential predictive value of RBP4 in the diagnosis and prognosis of CAD, additional investigations are warranted to further explore the underlying mechanism of RBP4 promoting the progression of CAD.

A few limitations of the study analysis are worth discussing. First, this analysis was a single-center study and only performed in Chinese population, and the findings should be cautiously generalized and further validated in other cohorts. Second, the study patients were all presented with stable CAD rather than acute coronary syndrome, and the sample size might be unable to reach statistical power, especially in subgroup analyses. Third, the observational finding of the associations between RBP4 and the prognosis in patients with stable CAD makes it impossible for us to investigate the causal relationship. Even though some confounders have been considered, we cannot rule out the possibility that unmeasured factors contribute to the observed associations.

## Conclusion

The higher circulating RBP4 levels were significantly associated with MACEs in patients with stable CAD. The finding should be further verified in multicenter studies with a larger sample.

## Data Availability Statement

The raw data supporting the conclusions of this article will be made available by the authors, without undue reservation.

## Ethics Statement

The studies involving human participants were reviewed and approved by the Ethics Committee Liyang People's Hospital. The identify information was removed before the data being released. The patients/participants provided their written informed consent to participate in this study.

## Author Contributions

KQ: conceptualization and project administration. XY: formal analysis. CX and YF: investigation. CX: methodology. YF: validation. KQ and XY: writing—original draft. MM: writing—review and editing. All authors contributed to the article and approved the submitted version.

## Conflict of Interest

The authors declare that the research was conducted in the absence of any commercial or financial relationships that could be construed as a potential conflict of interest.

## Publisher's Note

All claims expressed in this article are solely those of the authors and do not necessarily represent those of their affiliated organizations, or those of the publisher, the editors and the reviewers. Any product that may be evaluated in this article, or claim that may be made by its manufacturer, is not guaranteed or endorsed by the publisher.
