# Supplementary material for: Association Between Circulating Retinol-Binding Protein 4 and Adverse Cardiovascular Events in Stable Coronary Artery Disease
Source: Front Cardiovasc Med. 2022 Mar 17;9:829347. doi: 10.3389/fcvm.2022.829347 (PMC8968078; doi:10.3389/fcvm.2022.829347)
Supplement: Supplementary file 1 [file Data_Sheet_1.docx]

**Supplementary material**

**
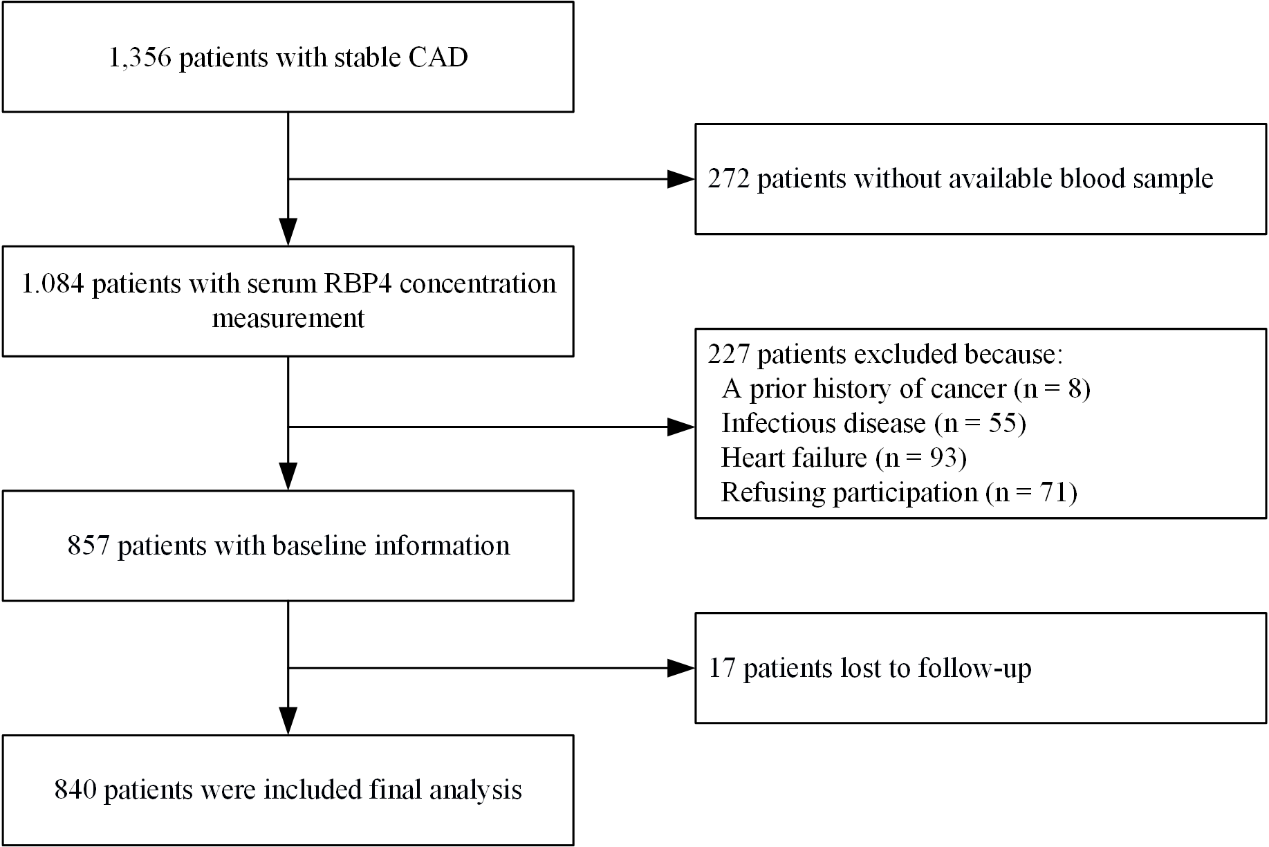
**

**Figure S1.** The flowchart of patients selection.

*CAD, coronary artery disease; RBP4, retinol-binding protein 4.*

**
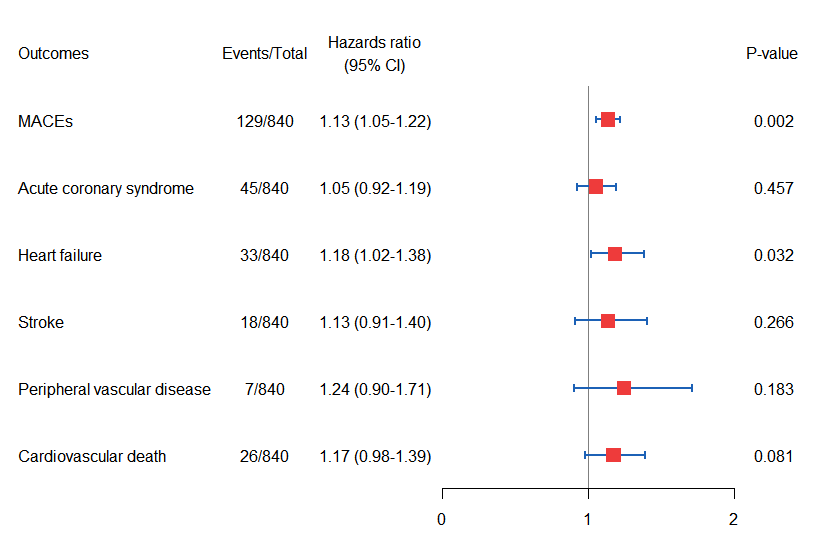
**

**Figure S2.** The forest plot of associations of RBP4 level and individual components of MACEs.

*The hazard ratios were calculated based on every 5 µg/mL increase of RBP4 level, adjusting for age, sex, body mass index, smoking, hypertension, diabetes mellitus, chronic kidney disease, and the usage of beta-blocker, statins, or ACEI/ARB.*

*RBP4, retinol-binding protein 4; MACEs, major adverse cardiovascular events; ACEI, angiotensin-converting enzyme inhibitor; ARB, angiotensin receptor blocker.*
